# Abrogation of Constitutive and Induced Type I and Type III Interferons and Interferon-Stimulated Genes in Keratinocytes by Canine Papillomavirus 2 E6 and E7

**DOI:** 10.3390/v12060677

**Published:** 2020-06-23

**Authors:** Sarah Quinlan, Susan May, Ryan Weeks, Hang Yuan, Jennifer A. Luff

**Affiliations:** 1 Department of Population Health and Pathobiology, North Carolina State University, Raleigh, NC 27607, USA; sfquinla@ncsu.edu (S.Q.); sepate@ncsu.edu (S.M.); rweeks@ucsd.edu (R.W.); 2 Department of Pathology, Georgetown University Medical Center, Washington, DC 20057, USA; Hang.Yuan@georgetown.edu

**Keywords:** canine, papillomavirus, interferon, interferon-stimulated gene, E6, E7

## Abstract

Cutaneous papillomaviruses can cause severe, persistent infections and skin cancer in immunodeficient patients, including people with X-linked severe combined immunodeficiency (XSCID). A similar phenotype is observed in a canine model of XSCID; these dogs acquire severe cutaneous papillomavirus infections that can progress to cancer in association with canine papillomavirus type 2 (CPV2). This canine model system provides a natural spontaneous animal model for investigation of papillomavirus infections in immunodeficient patients. Currently, it is unknown if CPV2 can subvert the innate immune system and interfere with its ability to express antiviral cytokines, which are critical in the host defense against viral pathogens. The aim of the current study was to determine if the oncogenes E6 and E7 from CPV2 interfere with expression of antiviral cytokines in keratinocytes, the target cells of papillomavirus infections. We determined that E6 but not E7 interferes with the constitutive expression of some antiviral cytokines, including interferon (IFN)-β and the IFN-stimulated gene IFIT1. Both E6 and E7 interfere with the transcriptional upregulation of the antiviral cytokines in response to stimulation with the dsDNA Poly(dA:dT). In contrast, while E6 also interferes with the transcriptional upregulation of antiviral cytokines in response to stimulation with the dsRNA Poly(I:C), E7 interferes with only a subset of these antiviral cytokines. Finally, we demonstrated that E7 but not E6 abrogates signaling through the type I IFN receptor. Taken together, CPV2 E6 and E7 both impact expression of antiviral cytokines in canine keratinocytes, albeit likely through different mechanisms.

## 1. Introduction

Papillomaviruses (PV) are a widely prevalent, heterogeneous group of host restricted and epitheliotropic viruses within the *Papillomaviridae* family [1,2]. They are circular, double stranded, DNA viruses, with a viral genome approximately 8 kb in length [1,2]. Over 300 types have been identified within mammals and bird species, with the majority being human isolates [1,2,3]. Human papillomaviruses (HPV) are classified into five genera, Alpha, Beta, Gamma, Mu, and Nu [1,2,3]. The alpha genus comprises those HPV types that infect the mucosal epithelium, and are divided into low-risk types, which cause benign genital warts, and high-risk types, which are associated with the development of cervical cancer as well as other anogenital and oropharyngeal cancers [1,2,3]. The Beta genus PVs are those that preferentially infect cutaneous sites causing benign papillomas or plaques [4,5,6]. However, cutaneous beta-PVs have been co-associated with ultraviolet (UV) radiation in the development of a subset of non-melanoma skin cancers (NMSC) [4,5,6]. A similar co-association between a feline cutaneous papillomavirus and UV exposure has been proposed for the development of cutaneous squamous cell carcinoma in cats [7]. The immune system is critical in controlling PV infections; thus, individuals with immunodeficiencies, such as those on immunosuppressive therapies due to organ transplantation, those infected with human immunodeficiency virus, or those with genetic immunodeficiencies, such as epidermodysplasia verruciformis (EV) or X-linked severe combined immunodeficiency (XSCID), are at a remarkably increased risk of developing persistent and severe cutaneous PV infections that commonly progress into NMSCs [4,5,6].

A similar increased risk of severe cutaneous PV infections has been observed in a research colony of dogs with XSCID [8]. These dogs are used as an animal model for human XSCID and exhibit a similar clinical and immunological phenotype as their human XSCID counterparts [9,10]. As with humans, these dogs require bone marrow transplants (BMT) within the first few weeks of life to survive. By 8 to 15 months post-BMT, ~70% of the dogs developed severe spontaneous cutaneous PV infections, and of those affected dogs, ~70% progressed to metastatic squamous cell carcinoma (SCC) [8]. Immunocompetent dogs in the same colony do not acquire these infections, suggesting that there is a remaining immune deficit in these dogs, which likely reflects what is happening with human XSCID patients [8]. Canine papillomavirus 2 (CPV2) is the virus associated with these cutaneous infections in XSCID dogs [11]. Because canine and human PVs share key biological characteristics and mechanisms of action, they are an ideal, natural model to study viral–host interactions of a cutaneous PV. In addition, the dog is a unique large animal model that can serve as a bridge for the translation of novel PV therapeutics into human testing.

Papillomaviruses mostly infect keratinocytes, key barrier skin cells that are capable of mounting an immune response by initiating expression of antiviral cytokines, such as type I and III interferons (IFNs) and IFN-stimulated genes, which act to eliminate the virus [12,13]. Keratinocytes recognize viral pathogens through expression of the pattern recognition receptors, such as the cytosolic nucleic acid sensors [14]. The cytosolic RNA receptors include melanoma differentiation associated gene 5 (MDA5) and retinoic acid-inducible gene I (RIGI) [15]. They can recognize double-stranded RNA (dsRNA) from RNA viruses, as well as dsRNA formed as an intermediate product of viral DNA replication [15]. Cytosolic DNA sensors include DNA-dependent activation of interferon regulatory factors (DAI) and interferon inducible gene 16 (IFI16) [16]. Upon recognition, these cytosolic nucleic acid sensors initiate signaling cascades leading to upregulation of type I and III IFNs [14]. In keratinocytes, this includes IFN-κ, IFN-β, IFN-λ1, and IFN-λ2/3 [17,18,19]. These IFNs are then released from the cell and bind to IFN receptors on the same or adjacent cells [14]. Binding to these IFN receptors initiates a second signaling cascade that culminates in the upregulation of hundreds of IFN-stimulated genes, which act to suppress and control infection [14], including IFN-Inducted Protein With Tetratricopeptide Repeats 1 (IFIT1), IFN regulatory factor 7 (IRF7), and DAI.

The mechanisms by which high-risk mucosal PVs abrogate the immune system are well studied. These high-risk mucosal PVs express oncogenes E6 and E7, which inhibit antiviral IFN and IFN-stimulated gene production through various mechanisms [20,21,22,23]. However, despite cutaneous PV’s significant effect on immunodeficient patients, little is known about their immune evasion strategies, but limited evidence suggests that cutaneous PV E6 and E7 may repress the antiviral response differently than mucosal PVs [24]. Using the canine cutaneous CPV2 model, we aimed to investigate the effect of cutaneous PV E6 and E7 on constitutive and induced mRNA expression of antiviral IFN and IFN-stimulated genes.

## 2. Materials and Methods

### 2.1. Plasmids and Retrovirus Transduction

Wild-type CPV2 E6 or E7 genes were each amplified from the viral genome by PCR and subcloned into the retrovirus vector pLSXN at the sites EcoRI and BamHI (Takara Bio USA Inc., Mountain View, CA, USA) as previously described [25]. Retrovirus stocks were prepared by transfecting the retrovirus packaging cell line SD3443 cells with vector only, CPV2 E6 or CPV2 E7 retrovirus constructs using Fugene (Roche applied science, Penzberg, Germany) as specified by the manufacturer and as previously described [25]. Culture supernatants containing retrovirus were collected after 48 h post-transfection and viral titers were determined using 3T3 cells. Canine primary epidermal keratinocytes (CPEKs; ZenBio, Research Triangle Park, NC, USA) were infected at a multiplicity of 10 PFU/cells with retrovirus containing vector only or wild type E6 or E7. Retrovirus-infected cells were selected in G418 (50 ng/mL) for two days.

### 2.2. Cell Culture

CPEKs were maintained in canine keratinocyte media CNT-09 (ZenBio) with the addition of penicillin–streptomycin antibiotics (Sigma-Aldrich, St. Louis, MO, USA). The pattern recognition receptor ligands used included Poly(I:C) high molecular weight complexed to Lyovec (InvivoGen, San Diego, CA, USA) and Poly(dA:dT) complexed to Lyovec (InvivoGen). Poly(I:C), when complexed to Lyovec, preferentially activates the cytosolic RNA sensors and not RNA sensors that reside within the endosomal compartment. Ligands were reconstituted with endotoxin free water as directed by the manufacturer. Recombinant IFN-β (PeproTech, Rocky Hill, NJ, USA) was reconstituted as recommended by the manufacturer. For stimulation experiments, CPEKs were routinely passaged and seeded into 6-well tissue culture plates at 3 × 10^5^ cells/mL in 2 mL complete media. The cells were incubated at 37 °C and 5% CO_2_ overnight. The media was then removed and replaced with 2 mL CNT-09 without antibiotics before stimulation with 250 ng/mL Poly(I:C), 750 ng/mL Poly(dA:dT), 5000 Units/mL IFN-β, or mock-stimulated with endotoxin free water that was used for reconstitution of the ligands. The cells were then incubated for an additional 24 h at 37 °C before the cells were lysed for RNA extraction.

### 2.3. Reverse Transcription Real Time PCR

Total RNA was extracted from cell lysates using a commercially available kit (RNeasy mini kit, Qiagen, Hilden, Germany). Complementary DNA (cDNA) was generated (QuantiTect, Qiagen, Hilden, Germany) from 1000 ng of total RNA following manufacturer’s recommended protocol. The resulting 20 μL cDNA was diluted with 180 μL RNase free water. Real time PCR using SYBR green detection (Qiagen) was performed for the following genes: Ribosomal Protein L13a (RPL13A), IFIT1, IFN-λ1, IFN-λ2, IFN-κ, IRF7, RIGI, MDA5, DAI, and IFI16. Primer pairs for DAI, MDA5, RIGI, IFI16, and IFN-κ have been previously published [26,27]. Primer pairs for RPL13A were designed based upon the GenBank sequence with accession number NM_001313766.1 using Primer 3 software (Whitehead Institute, Cambridge, MA, USA). Primer pairs for CPV2 E6 and CPV2 E7 were designed based upon the GenBank sequence with accession number NC_006564.1, using Primer 3 software. Primer pairs for IFN-λ1 and IFN-λ2 were designed based upon sequences generated from a study on the comparative genomics of IFN-λ, where three canine IFN-λ sequences were identified [28]. There are currently two different GenBank sequences listed for canine IFN-λ1. The GenBank sequence with accession number NM_001114853 corresponds with IFN-λ1 [28,29]; the GenBank sequence with accession number XM_014117025.2 most likely corresponds with IFN-λ2, although the exact nomenclature has not been designated and we will therefore refer to it here as IFN-λ2-like (IFN-λ2L). Primer pairs for IFN-λ1 and IFN-λ2L were designed based upon these sequences using Primer 3 software. Primer pairs for IRF7 were generated using the GenBank predicted sequence for canine IRF7 (GenBank accession number MT333743). Primer efficiencies were determined using a standard curve generated using purified PCR product. Primer sequences and efficiencies are listed in Table 1. Reaction mixtures contained 12.5 μL QuantiTech SYBR green master mix, 0.2 μM concentration of the forward and reverse primers, 10 μL of diluted cDNA (1:10), and enough RNase free water to yield a total of 25 μL reaction mixture. Real time RT-PCR for IFN-β and the reference gene Vacuolar Protein Trafficking and Biogenesis Associated (CCZ1) was performed using the validated Taqman Gene Expression Assay system (Applied Biosystems, Foster City, CA) with the following primer and probe sets following recommended protocols: IFN-β (Cf03644503_s1) and CCZ1 (Cf02643815.m1). All real time PCR reactions were performed in 96-well plates on the Roche 480 Light Cycler system.

Baseline expression of individual genes within vector only cells was normalized to expression of the reference gene (RPL13A for SYBR green and CCZ1 for Taqman assays) and graphed as the Delta Cq. To use Taqman IFN-β data in comparison with other genes in SYBR green assays, IFN-β results were normalized by normalizing the Taqman reference gene against the SYBR green reference gene (averaged across three experiments). To determine fold change between vector only and E6 or E7-expressing cells at baseline, the expression of individual genes was normalized to expression of the reference gene (RPL13A for SYBR green and CCZ1 for Taqman assays) and was calibrated to mRNA expression in vector only unstimulated cells based upon the 2^−ΔΔCq^ method [30]. To determine the fold change between vector only unstimulated and vector only stimulated cells, the expression of individual genes was normalized to the expression of the reference gene (RPL13A for SYBR green and CCZ1 for Taqman assays) and was calibrated to mRNA expression in vector only unstimulated cells based upon the 2^−ΔΔCq^ method [30]. To determine fold change (normalized to vector only mRNA expression), expression of individual genes was normalized to the expression of the reference gene (RPL13A for SYBR green and CCZ1 for Taqman assays) and was calibrated to mRNA expression in vector only unstimulated cells based upon the 2^−ΔΔCq^ method. Fold change in E6- or E7-expressing cells was then expressed as a ratio to fold change in vector only expressing cells to determine fold induction (normalized to vector only). To determine fold change (normalized to unstimulated cells mRNA expression), expression of individual genes was normalized to the expression of the reference gene (RPL13A for SYBR green and CCZ1 for Taqman assays) and was calibrated to mRNA expression of its own baseline unstimulated cells based upon the 2^−ΔΔCq^ method. Fold change in E6- or E7-expressing cells was then expressed as a ratio to fold change in vector only expressing cells to determine fold induction (each normalized to its own baseline).

### 2.4. Statistical Analysis

Statistical analysis and graphical presentation were performed using Graph Pad Prism 7 software (GraphPad software, San Diego, CA, USA). Mean values for relative gene expression were compared between unstimulated and stimulated samples or between vector only cells and CPV2 E6- or CPV2 E7-expressing cells using unpaired Student *t*-test. A *p*-value < 0.05 was considered significant.

## 3. Results

### 3.1. Constitutive mRNA Expression of a Subset of IFN and IFN-Stimulated Genes Is Reduced by CPV2 E6 in Canine Keratinocytes

**Figure 1:**
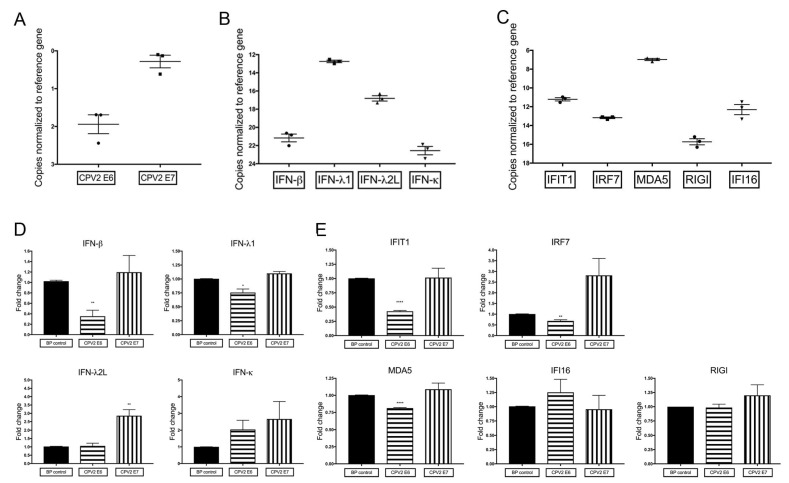
Diminished constitutive mRNA expression of antiviral cytokines in canine papillomavirus type 2 (CPV2) E6- but not E7-expressing canine keratinocytes. Primary canine keratinocytes expressing vector only, CPV2 E6, or CPV2 E7 were cultured for 48 h before mRNA expression of interferons (IFN) and IFN-stimulated genes were assessed by quantitative RT-PCR. (**A**) Expression levels of E6 and E7 in E6- or E7-expressing keratinocytes are graphed as the Delta Cq to compare expression levels of E6 and E7 within these experiments. The *y*-axis is reversed, so that the gene with the highest mRNA expression is positioned highest on the graph. Results are expressed as mean ± SEM of three independent experiments performed in duplicate. (**B**,**C**) Expression levels of IFNs (B) or IFN-stimulated genes (C) in vector only cells are graphed as the Delta Cq to show the comparative expression levels of each IFN or IFN-stimulated gene. The *y*-axis is reversed, so that the gene with the highest mRNA expression is positioned highest on the graph. Results are expressed as mean ± SEM of three independent experiments performed in duplicate. (**D**,**E**) Resulting Cq values for vector only, CPV2 E6-expressing, and CPV2 E7-expressing cells were normalized to the Cq value of the reference gene and calibrated to mRNA expression in vector only cells. mRNA expression of IFN-β, IFN-λ1, IFIT1, IRF7, and MDA5 was diminished in canine keratinocytes expressing CPV2 E6. Expression of CPV2 E7 in keratinocytes did not result in any decrease in IFN or IFN-stimulated gene expression, although expression of IFN-λ2L and IRF7 were slightly increased. Experiments were performed in duplicate and repeated in three independent experiments. Results are expressed as mean ± SEM. * *p* < 0.05; ** *p* < 0.01; **** *p* < 0.0001. IFIT1, IFN-Induced Protein with Tetratricopeptide Repeats 1; IRF7, Interferon regulatory factor 7; MDA5, melanoma differentiation associated gene 5; RIGI, retinoic acid-inducible gene I; and IFI16, interferon inducible gene 16.

Certain high-risk mucosal human papillomaviruses are able to evade immune detection by interfering with constitutive expression of antiviral cytokines [22,31]. To investigate if a similar effect is seen for CPV2, canine keratinocytes expressing E6 or E7 genes were generated using retrovirus transduction. CPV2 E6 and E7 expression was confirmed using real time reverse transcriptase PCR (RT-qPCR) and conventional RT-PCR, as antibodies to detect these proteins are not available. RT-qPCR results of E6 and E7 expression are presented in Figure 1A. Cq values averaged 16.6 for CPV2 E6 (reference gene Cq values averaging 14.7) in CPV2 E6-expressing keratinocytes, and Cq values averaged 14.9 for CPV2 E7 (reference gene Cq values averaging 14.6) in CPV2 E7-expressing keratinocytes. Given the similar primer efficiencies for E6 and E7 (Table 1), this demonstrates an ~3-fold difference between expression of E6 versus E7. While both genes are adequately expressed, with Cq values close to the level of the reference gene, it precludes direct comparison of results between E6 and E7. Conventional RT-PCR results are provided as a supplemental figure (Supplemental Figure S1). These keratinocytes were then cultured and expression levels of several antiviral cytokines were evaluated, including the type I IFNs (IFN-β and IFN-κ), type III IFNs (IFN-λ1 and IFN-λ2L), IFN-stimulated genes (IFIT1 and IRF7), and the pattern recognition receptors (IFI16, MDA5, and RIGI). IFN-α and the IFN-stimulated gene DAI were not examined, as expression levels were too low to accurately assess. Normal canine keratinocytes expressing vector only, CPV2 E6, or CPV2 E7 were cultured for 48 h before evaluation of mRNA expression levels. To compare the expression levels of the different IFNs and IFN-stimulated genes, we graphed the Delta Cq of each gene in vector only cells (Figure 1B,C). IFN-λ1 expression was highest, followed by IFN-λ2L, and then IFN-β and IFN-κ (Figure 1B). For the IFN-stimulated genes and pattern recognition receptors, expression of MDA5 was highest, followed by IFIT1, IFI16, IRF7, and RIGI (Figure 1C). In the canine keratinocytes that expressed CPV2 E6 or E7, we found that CPV2 E6, but not E7, reduced mRNA expression of IFN-β, IFN-λ1, IFIT1, IRF7, and MDA5 (Figure 1D,E). The expression of both IFN-β and IFIT1 was reduced by more than 50% compared to cells expressing vector only. CPV2 E6 did not alter expression levels of IFN-λ2L, IFN-κ, nor the pattern recognition receptors RIGI and IFI16. E7 did not diminish expression of any of the examined IFN or IFN-stimulated genes, but there was a 2–3-fold increase in expression of IFN-λ2L and IRF7. Although neither CPV2 E6 nor CPV2 E7 significantly altered expression of IFN-κ, there was a slight upregulation in both; we considered the possibility of a cumulative effect of E6 and E7 on IFN-κ expression. To address this possibility, we repeated this experiment using canine keratinocytes that expressed both E6 and E7 and found that IFN-κ was not significantly upregulated (Supplemental Figure S2). We also included IFIT1, which was decreased in E6- and E7-expressing keratinocytes similarly to the E6 only expressing cells (Supplemental Figure S2). These results suggest that E6 but not E7 reduces constitutive mRNA expression of a subset of IFNs and IFN-stimulated genes.

### 3.2. Diminished Poly(dA:dT)-Induction of IFN and IFN-Stimulated Genes by CPV2 E6 and E7 in Canine Keratinocytes

**Figure 2:**
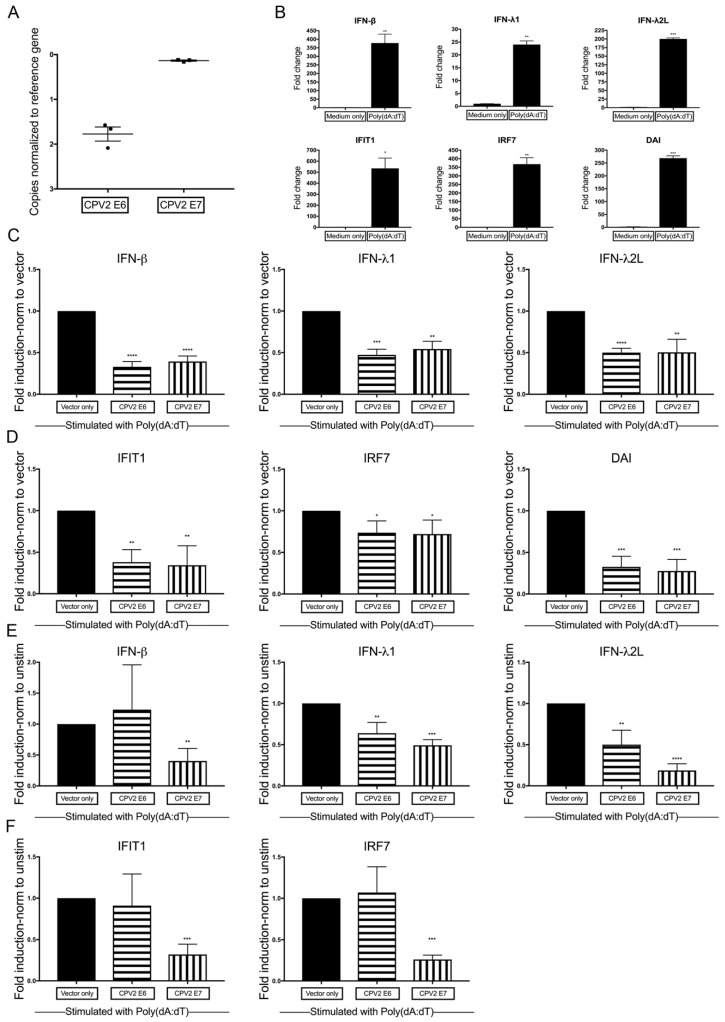
Diminished dsDNA-induction of antiviral cytokines in CPV2 E6- and E7-expressing keratinocytes. Primary canine keratinocytes expressing vector only, CPV2 E6, or CPV2 E7 were stimulated with the dsDNA ligand Poly(dA:dT). mRNA expression of interferons (IFN) and IFN-stimulated genes was assessed 24 h after stimulation by quantitative RT-PCR. (**A**) Expression levels of E6 and E7 in E6- or E7-expressing keratinocytes are graphed as the Delta Cq to compare expression levels of E6 and E7 within these experiments. The *y*-axis is reversed, so that the gene with the highest mRNA expression is positioned highest on the graph. Results are expressed as mean ± SEM of three independent experiments performed in duplicate. (**B**) In vector only expressing cells, resulting Cq values were normalized to the Cq value of the reference gene and calibrated to mRNA expression in unstimulated cells. Results are expressed as mean ± SD of one representative independent experiment of three performed in duplicate. (**C**,**D**) In vector only and CPV2 E6- or E7-expressing cells, resulting Cq values were normalized to the Cq value of the reference gene and calibrated to mRNA expression in vector only stimulated cells; fold induction was calculated as a ratio (fold change E6 or E7 cells/fold change vector only cells). Experiments were performed in duplicate and repeated in three independent experiments. Results are expressed as the mean ± SD. (**E**,**F**) In vector only and CPV2 E6- or E7-expressing cells, resulting Cq values were normalized to the Cq value of the reference gene and calibrated to its own mRNA expression at baseline; fold induction was calculated as a ratio (fold change E6 or E7 cells/fold change vector only cells). Experiments were performed in duplicate and repeated in three independent experiments. Results are expressed as the mean ± SD. * *p* < 0.05; ** *p* < 0.01; *** *p* < 0.001; **** *p* < 0.0001. IFIT1, IFN-Induced Protein with Tetratricopeptide Repeats 1; IRF7, IFN regulator factor 7; DAI, DNA-dependent activation of interferon regulatory factors.

Some high-risk HPVs abrogate signaling through the pattern recognition receptors to diminish upregulation of antiviral cytokines [20,21,22,23]. To determine if CPV2 E6 or E7 impacts IFN and IFN-stimulated gene expression after stimulation of the DNA-sensing pathway, E6 and E7 were expressed in canine keratinocytes and stimulated with poly(dA:dT), a synthetic analog of dsDNA that stimulates the DNA-sensing nucleic acid sensors. Normal canine keratinocytes expressing vector only, CPV2 E6, or CPV2 E7 were cultured for 24 h, stimulated with poly(dA:dT) or water control for an additional 24 h before evaluation of mRNA expression. Expression of CPV2 E6 and E7 mRNA within E6- or E7-expressing keratinocytes was confirmed using RT-qPCR (Figure 2A). Cq values averaged 16.4 for CPV2 E6 (reference gene Cq values averaging 14.6) in CPV2 E6-expressing keratinocytes, and Cq values averaged 14.8 for CPV2 E7 (reference gene Cq values averaging 14.7) in CPV2 E7-expressing keratinocytes, which demonstrates an ~3-fold difference between expression of E6 versus E7, as seen above. While both are adequately expressed, with Cq values close to the level of the reference gene, it precludes direct comparison of results between E6 and E7. We next demonstrated that each gene was significantly upregulated in stimulated cells compared with unstimulated cells in vector only control cells (Figure 2B).

We then wanted to determine whether CPV2 E6 or E7 impacted the total expression of IFNs and IFN-stimulated genes compared with expression in vector only keratinocytes after dsDNA stimulation. As the fold induction between experiments varied, averaging fold induction across experiments was meaningless. Therefore, since our question was on the impact of E6 and E7 on IFN and IFN-stimulated gene expression compared to vector only cells, we expressed the fold change in mRNA as a ratio (fold change E6 or E7/fold change vector only) to determine the fold induction. By normalizing to expression in vector only cells, the results will demonstrate the total expression of IFNs or IFN-stimulated genes in E6 and E7 cells compared with vector only cells. Canine keratinocytes that expressed CPV2 E6 or E7 had significantly reduced mRNA expression of IFN-β, IFN-λ1, IFN-λ2L, IFIT1, and DAI (Figure 2C,D) after stimulation with dsDNA. The impact on IRF7 upregulation was not as large, but it was still significantly decreased compared with vector only cells. Neither IFN-κ nor IFN-α were included, as they were not significantly upregulated after stimulation ([27] and data not shown).

Given that CPV2 E6 and E7 significantly reduced mRNA expression of some IFNs and IFN-stimulated genes at baseline, we wanted to determine any additional impact of CPV2 E6 and E7 on the dsDNA-induced expression of IFNs and IFN-stimulated genes. To determine this, the same data used to generate Figure 2C,D were reanalyzed and normalized to its own baseline (unstimulated) and then expressed as a ratio (fold change E6 or E7/fold change vector only) to determine the fold induction. These results demonstrate the impact of E6 and E7 on induced expression of IFNs and IFN-stimulated genes. CPV2 E6 significantly impacts induced expression of IFN-λ1 and IFN-λ2L but not IFN-β, IFIT1, nor IRF7 (Figure 2E,F), whereas CPV2 E7 significantly impacts induced expression of IFN-β, IFN-λ1, IFN-λ2L, IFIT1, and IRF7. DAI was not analyzed in this manner, as baseline data for DAI was at the limit of detection for all samples.

### 3.3. Diminished Poly(I:C)-Induction of IFN and IFN-Stimulated Genes by CPV2 E6 and E7 in Canine Keratinocytes

**Figure 3:**
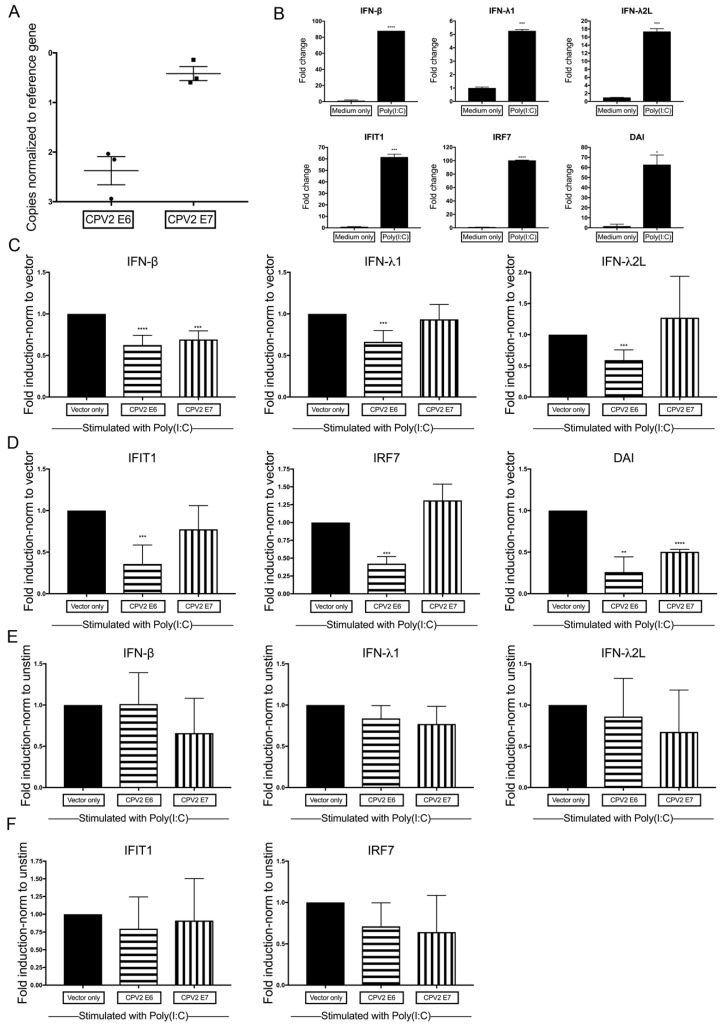
Diminished dsRNA-induction of antiviral cytokine in CPV2 E6- and E7-expressing keratinocytes. Primary canine keratinocytes expressing vector only, CPV2 E6, or CPV2 E7 were stimulated with the dsRNA ligand Poly(I:C). mRNA expression of interferons (IFN) and IFN-stimulated genes was assessed 24 h after stimulation by quantitative RT-PCR. (**A**) Expression levels of E6 and E7 in E6- or E7-expressing keratinocytes are graphed as the Delta Cq to compare expression levels of E6 and E7. The *y*-axis is reversed, so that the gene with the highest mRNA expression is positioned highest on the graph. Results are expressed as mean ± SEM of three independent experiments performed in duplicate. (**B**) In vector only expressing cells, resulting Cq values were normalized to the Cq value of the reference gene and calibrated to mRNA expression in unstimulated cells. Results are expressed as mean ± SD of one representative independent experiment of five performed in duplicate. (**C**,**D**) In vector only and CPV2 E6- or E7-expressing cells, resulting Cq values were normalized to the Cq value of the reference gene and calibrated to mRNA expression in vector only stimulated cells; fold induction was calculated as a ratio (fold change E6 or E7 cells/fold change vector only cells). Experiments were performed in duplicate and repeated in three independent experiments. Results are expressed as the mean ± SD. (**E**,**F**) In vector only and CPV2 E6- or E7-expressing cells, resulting Cq values were normalized to the Cq value of the reference gene and calibrated to its own mRNA expression at baseline; fold induction was calculated as a ratio (fold change E6 or E7 cells/fold change vector only cells). Experiments were performed in duplicate and repeated in five independent experiments. Results are expressed as the mean ± SD. * *p* < 0.05; ** *p* < 0.01; *** *p* < 0.001; **** *p* < 0.0001. IFIT1, IFN-Induced Protein with Tetratricopeptide Repeats 1; IRF7, IFN regulator factor 7; DAI, DNA-dependent activation of interferon regulatory factors.

To help determine if E6 and E7 also affects the dsRNA signaling pathway, E6 and E7 were expressed in canine keratinocytes and stimulated with poly(I:C), a synthetic analog of dsRNA that stimulates the cytosolic RNA-sensors MDA5 and RIGI. Normal canine keratinocytes expressing vector only, CPV2 E6, or CPV2 E7 were cultured for 24 h, stimulated with poly(I:C) or water control for an additional 24 h before evaluation of mRNA expression. Expression of CPV2 E6 and E7 mRNA within E6- or E7-expressing keratinocytes was confirmed using RT-qPCR (Figure 3A). Cq values averaged 17.3 for CPV2 E6 (reference gene Cq values averaging 14.9) in CPV2 E6-expressing keratinocytes, and Cq values averaged 15.3 for CPV2 E7 (reference gene Cq values averaging 14.9) in CPV2 E7-expressing keratinocytes, which demonstrates an ~3–4-fold difference between expression of E6 versus E7. While both were adequately expressed, with Cq values close to the level of the reference gene, it precludes direct comparison of results between E6 and E7, as noted above. We next demonstrated that each gene was significantly upregulated in stimulated cells compared with unstimulated cells in vector only cells (Figure 3B).

We then wanted to determine if CPV2 E6 or E7 impacted the total expression of IFNs and IFN-stimulated genes compared with expression in vector only keratinocytes after dsRNA stimulation. As above for dsDNA (Section 3.2), we expressed the fold change in mRNA as a ratio (fold change E6 or E7/fold change vector only) to determine the fold induction. By normalizing to the expression in vector only cells, the results demonstrate the total expression of IFNs or IFN-stimulated genes in E6 and E7 cells compared to vector only cells after stimulation with dsRNA. Canine keratinocytes that expressed CPV2 E6 had significantly reduced mRNA expression of the IFNs (IFN-β, IFN-λ1, and IFN-λ2L) and IFN-stimulated genes (IFIT1, IRF7, and DAI), while cells that expressed CPV2 E7 had significantly reduced mRNA upregulation of only IFN-β and DAI (Figure 3C,D).

Similar to above (Section 3.2), we wanted to determine any additional impact of CPV2 E6 and E7 on the dsRNA-induced expression of IFNs and IFN-stimulated genes. To this aim, the same data used to generate Figure 3C,D were reanalyzed and normalized to its own baseline (unstimulated) and then expressed as a ratio (fold change E6 or E7/fold change vector only) to determine the fold induction. These results demonstrate the impact of E6 and E7 on dsRNA-induced expression of IFNs and IFN-stimulated genes. Neither CPV2 E6 nor E7 impacts induce expression of IFNs or IFN-stimulated genes after dsRNA stimulation. However, the error bars are fairly large, which may obscure subtle impacts on fold induction. It is possible, for example, that CPV2 E7 impacts induce expression of IFN-β, as total expression of IFN-β compared to vector only was decreased (Figure 3C), and CPV2 E7 did not inhibit baseline IFN-β expression. As above, DAI was not analyzed in this manner, as baseline data for DAI was at the limit of detection for all samples.

### 3.4. Diminished IFN-β-Induction of IFN-Stimulated Genes by CPV2 E7 but Not E6 in Canine Keratinocytes

**Figure 4:**
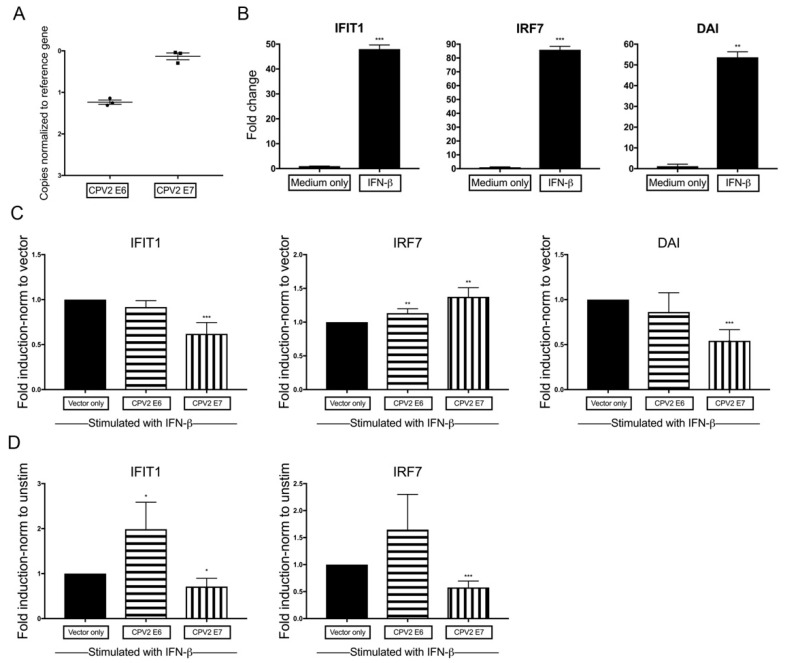
Diminished signaling through the type I IFN receptor by CPV2 E7- but not E6-expressing keratinocytes. Primary canine keratinocytes expressing vector only, CPV2 E6, or CPV2 E7 were stimulated with IFN-β. IFN-stimulated gene mRNA expression was assessed 24 h after stimulation by quantitative RT-PCR. (**A**) Expression levels of E6 and E7 in E6- or E7-expressing keratinocytes are graphed as the Delta Cq to compare expression levels of E6 and E7. The *y*-axis is reversed, so that the gene with the highest mRNA expression is positioned highest on the graph. Results are expressed as mean ± SEM of three independent experiments performed in duplicate. (**B**) In vector only expressing cells, resulting Cq values were normalized to the Cq value of the reference gene and calibrated to mRNA expression in unstimulated cells. Results are expressed as mean ± SD of one representative independent experiment of four performed in duplicate. (**C**) In vector only and CPV2 E6- or E7-expressing cells, resulting Cq values were normalized to the Cq value of the reference gene and calibrated to mRNA expression in vector only stimulated cells; fold induction was calculated as a ratio (fold change E6 or E7 cells/fold change vector only cells). Experiments were performed in duplicate and repeated in four independent experiments. Results are expressed as the mean ± SD. (**D**) In vector only and CPV2 E6- or E7-expressing cells, resulting Cq values were normalized to the Cq value of the reference gene and calibrated to its own mRNA expression at baseline; fold induction was calculated as a ratio (fold change E6 or E7 cells/fold change vector only cells). Experiments were performed in duplicate and repeated in four independent experiments. Results are expressed as the mean ± SD. * *p* < 0.05; ** *p* < 0.01; *** *p* < 0.001. IFIT1, IFN-Induced Protein with Tetratricopeptide Repeats 1; IRF7, IFN regulator factor 7; DAI, DNA-dependent activation of interferon regulatory factors.

To determine whether the impact of E6 and E7 on IFN-stimulated gene expression is due to decreased signaling through the type I IFN receptor, canine keratinocytes were stimulated directly with IFN-β. To this aim, normal canine keratinocytes expressing vector only, CPV2 E6, or CPV2 E7 were cultured for 24 h, stimulated with IFN-β or unstimulated control for an additional 24 h before evaluation of mRNA expression levels of IFN-stimulated genes. Expression of CPV2 E6 and E7 mRNA within E6- or E7-expressing keratinocytes was confirmed using RT-qPCR (Figure 4A). Cq values averaged 16.0 for CPV2 E6 (reference gene Cq values averaging 14.8) in CPV2 E6-expressing keratinocytes, and Cq values averaged 15.1 for CPV2 E7 (reference gene Cq values averaging 14.9) in CPV2 E7-expressing keratinocytes, which demonstrates an ~3–4-fold difference between expression of E6 versus E7. While both were adequately expressed, with Cq values close to the level of the reference gene, it precludes direct comparison of results between E6 and E7, as noted above.

We then demonstrated that each gene was significantly upregulated in stimulated cells compared with unstimulated cells in vector only control cells (Figure 4B). As above for dsDNA (Section 3.2) and dsRNA (Section 3.3), we expressed the fold change in mRNA as a ratio (fold change E6 or E7/fold change vector only) to determine the fold induction. By normalizing to expression in vector only cells, the results demonstrate the total expression of IFNs or IFN-stimulated genes in E6 and E7 cells compared with vector only cells after stimulation with IFN-β. While canine keratinocytes expressing CPV2 E6 did not decrease expression of the IFN-stimulated genes, keratinocytes expressing CPV2 E7 significantly diminished expression of IFIT1 and DAI (Figure 4C). Unexpectedly, IRF7 expression was slightly increased in cells expressing either CPV2 E6 or E7 compared with vector only cells. The cause for the slight increase in IRF7 is unknown.

Similar to above (Section 3.2 and Section 3.3), we wanted to determine any additional impact of CPV2 E6 and E7 on the IFN-β-induced expression of IFN-stimulated genes. To determine this, the same data used to generate Figure 4C was reanalyzed and normalized to its own baseline (unstimulated) and then expressed as a ratio (fold change E6 or E7/fold change vector only) to determine the fold induction. These results demonstrate the impact of E6 and E7 on IFN-β-induced expression of IFN-stimulated genes. Induced expression of IFIT1 and IRF7 was increased in CPV2 E6-expressing keratinocytes, although only reaching significance for IFIT1, which reflects the initial lower baseline expression of IFIT1 and IRF7, which was overcome after stimulation with IFN-β (Figure 4D). In contrast, induced expression of IFIT1 and IRF7 was inhibited in CPV2 E7-expressing cells (Figure 4D). As above, DAI was not analyzed in this manner, as baseline data for DAI was at the limit of detection for all samples. These data indicate that CPV2 E7 but not E6 impacts the IFN-stimulated gene induced upregulation by the IFN-receptor signaling pathway.

## 4. Discussion

Many oncogenic viruses have evolved mechanisms to avoid recognition by the innate immune system, which enables the virus to persist in the host, and can ultimately lead to cancer [3,32,33]. Mucosal human PV (HPV) are causally associated with virtually all cases of cervical cancer, as well as a subset of other genital and head and neck cancers [34]. They express the oncogenes E6 and E7, which in addition to dysregulating tumor suppressor genes, can subvert the innate immune system by preventing upregulation of antiviral cytokines [3]. Cutaneous HPVs cause debilitating infections in immunodeficient people, which can progress to cancer at solar exposed sites [5,6]. It is likely that cutaneous HPV E6 and E7 are also able to interfere with innate immune signaling, although the underlying mechanisms are not as well established; however, limited studies suggest alternative mechanisms compared with mucosal PVs [24]. We were interested in determining whether the cutaneous CPV2 E6 and E7 can also interfere with innate immune signaling; this virus causes debilitating infections and metastatic cancers in an important animal model for human XSCID patients. The ability of CPV2, or any canine PV, to subvert immune signaling has been previously unknown. We have shown in this current study that, similar to HPVs, both CPV2 E6 and E7 can interfere with transcriptional upregulation of antiviral cytokines, likely affecting different parts of the innate signaling pathway.

**Figure 5:**
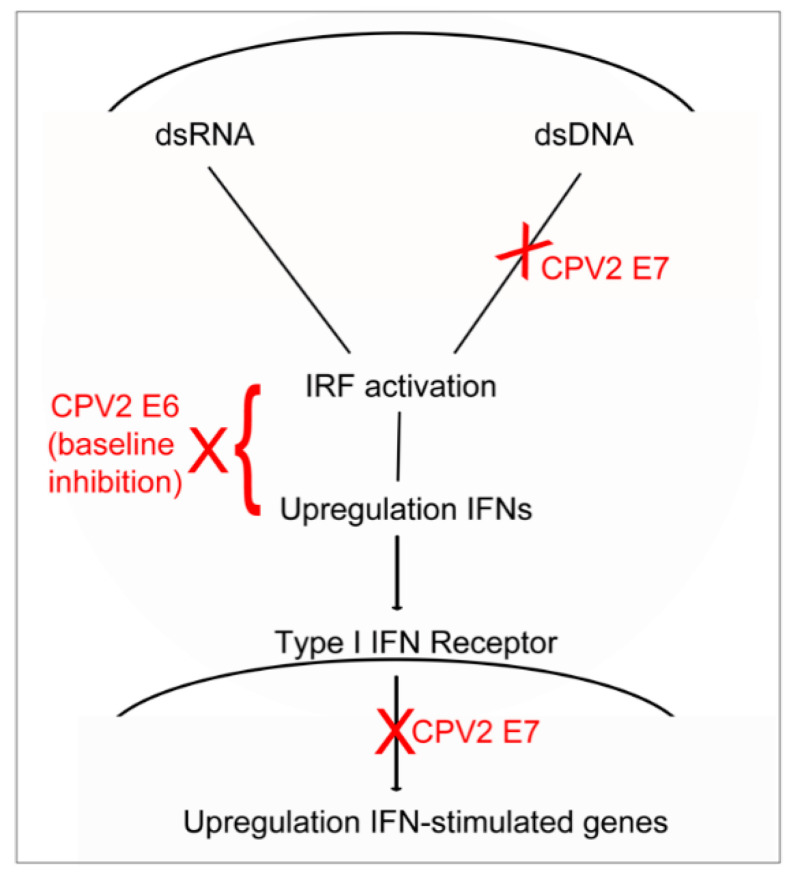
Proposed model of CPV2 E6 and E7 interference within the cytosolic dsDNA and dsRNA signaling pathways within keratinocytes. CPV2 E6 predominantly inhibits the basal expression of type I and III IFNs and IFN-stimulated genes, which ultimately results in diminished expression of IFNs and IFN-stimulated genes after stimulation with dsDNA and dsRNA. CPV2 E7 does not affect basal expression of IFNs and IFN-stimulated genes, but significantly impacts the induced IFN and IFN-stimulated gene expression in response to dsDNA stimulation. CPV2 E7 also abrogates signaling through the type I IFN receptor pathway to diminish expression of IFN-stimulated genes. CPV2, canine papillomavirus 2; IRF, interferon regulatory factor; IFN, interferon.

The E6 oncogenes from both mucosal and cutaneous PVs can repress IFN and IFN-stimulated gene expression. Dysfunction of IRFs by E6 is a well-known evasion technique utilized by the high-risk mucosal HPVs. E6 from the high-risk mucosal HPV16 can bind IRF3 and repress IFN-stimulated gene expression [23]. In contrast, E6 from a cutaneous HPV is unable to bind to IRF3 and only weakly antagonizes its activity [23,35]. Interestingly, E6 from another human cutaneous PV is critical for down-regulation of IRF1 [24], a function attributed to E7 of the mucosal PVs [21,36]; the underlying mechanism for IRF1 dysfunction, however, is not known. We found in the current study that CPV2 E6 diminishes constitutive expression of a subset of IFNs and IFN-stimulated genes, which ultimately impacts the final amount of IFN and IFN-stimulated gene expression compared to expression in vector only cells after stimulation with dsDNA and dsRNA. CPV2 E6 has a smaller impact on the dsDNA-induced (relative to baseline) upregulation of a subset of IFNs (type III IFNs) and IFN-stimulated genes (DAI) and no significant impact on the dsRNA-induced (relative to baseline) upregulation of IFNs or IFN-stimulated genes, with the exception of DAI, which is essentially not expressed in unstimulated cells. This suggests that the main impact of CPV2 E6 on basal expression of IFNs and IFN-stimulated genes ultimately effects expression of these genes within both the dsRNA and dsDNA sensing pathways; given this similar impact, E6 most likely interferes with a region of this shared pathway, such as activation or effector functions of the IFN regulatory factors (IRFs; Figure 5).

Global transcriptome analyses of cells harboring human mucosal PVs or their E6 oncogenes have demonstrated decreased expression of IFN-stimulated genes [22,31]. This was attributed to decreased constitutive expression of IFN-κ, resulting in decreased expression of both IFN-stimulated genes and pattern recognition receptors required for expression of inducible IFNs [22]. In the current study, we found that CPV2 E6 similarly repressed IFN and IFN-stimulated gene expression; however, the underlying mechanism is likely different from that of human mucosal PV E6, as CPV2 E6 did not alter expression of IFN-κ and pattern recognition receptors were only mildly decreased, if at all.

Similar to E6, the E7 oncogene of the mucosal HPVs can modulate the antiviral response through several mechanisms; studies on antiviral evasion strategies mediated by E7 from cutaneous PVs, however, are sparse. E7 from the high-risk mucosal HPVs represses IRF1 activity, leading to decreased IFN-β gene expression [21,36,37]. Both high-risk mucosal HPV16 and HPV18 antagonize the adaptor protein within the dsDNA signaling pathway Stimulator of IFN genes (STING) to repress signaling through the cytosolic DNA receptor pathway, although they do this through different mechanisms [20,38]. Further, HPV18 E7 can epigenetically silence RIGI, the DNA sensor cyclic GMP-AMP synthase (cGAS), and STING preventing upregulation of IFN and IFN-stimulated genes activated by dsRNA and dsDNA [39]. We found in this current study that the main impact of CPV2 E7 was on the dsDNA-induced expression of IFN and IFN-stimulated genes. E7 did not significantly diminish constitutive expression of antiviral cytokines, nor did it impact expression of IFNs or IFN-stimulated genes after stimulation with dsRNA, with the exception of IFN-β and DAI. This suggests that, given the main impact of CPV2 E7 on dsDNA- but not dsRNA-induced expression, E7 effects the dsDNA pathway upstream from the shared pathway with dsDNA, such as antagonism of STING or cGAS or another dsDNA sensor (Figure 5). We also unexpectedly found that expression of CPV2 E7 caused a slight upregulation of the constitutive expression of IFN-λ2L and IRF7, although the effect on IRF7 did not reach significance. The cause and biological significance of this is unknown, but it may reflect a direct or indirect activation of part of the antiviral signaling pathway.

We additionally examined signaling through the type I IFN receptor, which can be inhibited by both HPV E6 and E7 from the mucosal high-risk types [40,41]. HPV16 E7 can repress signaling by bind to IRF9, a key IFN regulatory component of the type I receptor pathway [40]. HPV18 E6 can bind to tyrosine-protein kinase 2 (TYK2) preventing phosphorylation of downstream signaling components [41]. In the current study, we found that E7 diminished signaling through the type I IFN receptor, suggesting E7 interferes with some component of this pathway (Figure 5). In contrast, however, CPV2 E6 did not inhibit signaling through the type 1 IFN receptor. Unexpectedly, both CPV2 E6 and E7 caused increased upregulation in the total amount of IRF7 compared to vector only cells. The IFN-β-induced expression (compared to baseline) was decreased only for CPV2 E7, reflecting the difference in the effect of E6 and E7 on baseline IRF7 expression. This higher expression of IRF7 may reflect a distinct effect of E6 and E7 on IRF7 induction that does not involve signaling through the type I IFN receptor. This could be a direct induction of IRF7 transcription by the viral proteins as can occur with Epstein–Barr virus, or an indirect effect such as induction of TNF-α or modulation of chromosomal accessibility, both which can cause upregulation of IRF7 [42]. It is also plausible that expression of both E6 and E7 together within keratinocytes could have a cumulative or magnified effect on IRF7 upregulation. However, additional studies are required to ascertain any cumulative effect of viral oncogenes on IRF7.

The underlying mechanisms by which cutaneous E6 and E7 interfere in these pathways have yet to be elucidated. However, as indicated above, there are many known mechanisms as to how mucosal HPV types interfere with the IFN and IFN-stimulated gene signaling to avoid immune detection, and it is likely mucosal and cutaneous types, including canine PVs, share some mechanisms and differ in others. Results of this study provide the foundation for future investigations to determine the mechanisms by which CPV2 E6 and E7 antagonize the IFN signaling pathways. Taken together, while CPV2 E6 and E7 both interfere with the antiviral signaling pathways, they likely accomplish this through different mechanisms. Determining these mechanisms can identify particular targets of cutaneous E6 and E7 that can be utilized to develop treatments to combat these oncogenic effects.

## 5. Conclusions

CPV2 E6 and E7 both impact expression of the type I and III IFNs and IFN-stimulated genes in canine keratinocytes, albeit likely through different mechanisms.

## Figures and Tables

**Table 1:** Primer sets and primer efficiency for reverse transcriptase real time PCR (RT-qPCR).

Target Gene	Primer Sequence Forward and Reverse (5′–3′)	Efficiency (%)
RPL13A (reference gene)	TGGGCCGGAAGGTTGTAGTCGT	99
	TTGCGGAGGAAGGCCAGGTAATTCA	
IFN-λ1	TCCCTACTTCCAAACCCACC	95
	GTTCTTCCAGGAGAGCGACT	
IFN-λ2L	CGCCTCTTCCCTAGAAACCGGGACC	96
	CTCCAGGACCTTCAGTGTCAAGGCC	
IRF7	GCAAGGTCTACTGGGAGGTG	97
	GTGCTGAAGTCGAAGATGGGG	
CPV2 E6	ATATTTATGAAACCGTTAGCC	99
	CGCAGCTGTCACAAGTGTTCC	
CPV2 E7	ACAGAGAGAACCTGGGCGATA	100
	ATAATGCCAAGCCCGTCTAA	

RPL13A, ribosomal protein L13a; IFN, interferon; IRF, interferon regulatory factor; CPV2, canine papillomavirus 2.

## Supplementary Materials

The following are available online at https://www.mdpi.com/1999-4915/12/6/677/s1, Figure S1: RT-PCR for canine papillomavirus 2 in canine keratinocytes; Figure S2: Baseline expression of interferon (IFN)-κ and IFN-Induced Protein with Tetratricopeptide Repeats 1 (IFIT1) in canine keratinocytes expressing canine papillomavirus (CPV)2 E6/E7.
