# Supplementary material for: Abrogation of Constitutive and Induced Type I and Type III Interferons and Interferon-Stimulated Genes in Keratinocytes by Canine Papillomavirus 2 E6 and E7
Source: Viruses. 2020 Jun 23;12(6):677. doi: 10.3390/v12060677 (PMC7354437; doi:10.3390/v12060677)
Supplement: Supplementary file 1 [file viruses-12-00677-s001.zip › viruses-809279 final supplementary/Figure S1.pdf]

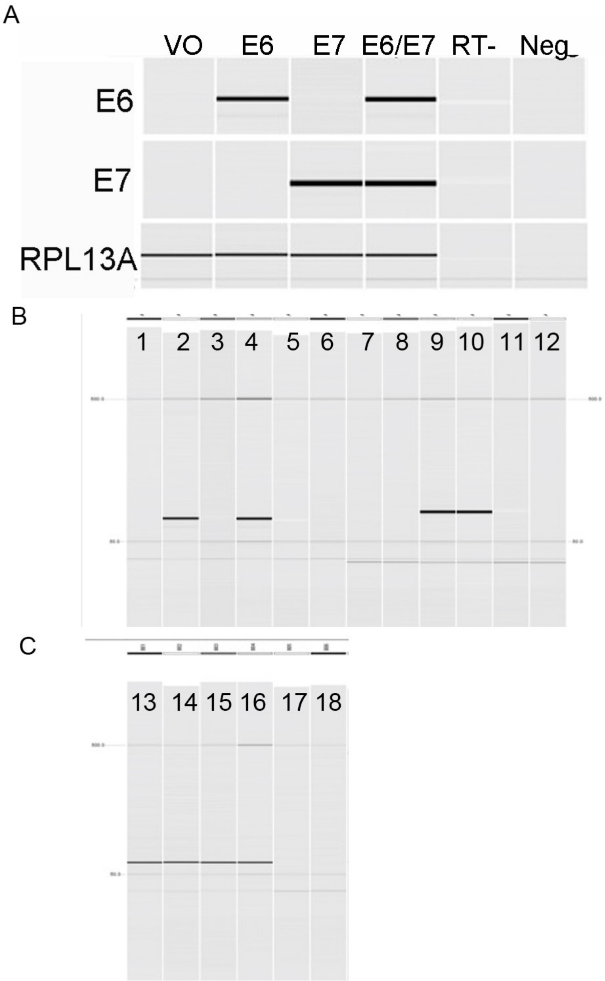

Supplemental Figure 1. A. RT-PCR for canine papillomavirus 2 (CPV2) E6 and E7 and RPL13A (reference gene) demonstrates mRNA expression within canine keratinocyte cell cultures. Total RNA was extracted from cell lysates from Vector only (VO), CPV2 E6, CPV2 E7, and CPV2 E6 and E7 expressing keratinocytes. cDNA was then generated from the total RNA, followed by conventional PCR for CPV2 E6, CPV2 E7, and RPL13A. The PCR products were size separated and visualized using an eGene HAD-GT12 capillary electrophoresis analyzer (renamed QIAxcel, Qiagen, Valencia, CA, USA). VO, vector only; RT-, reverse transcriptase negative; Neg, negative control. B and C. Raw images of gel run. Lanes 1-6 using primer to detect CPV2 E6; Lanes 7-12 using primer to detect CPV2 E7; Lanes 13-18 using primer to detect RPL13A. Lanes 1, 7, and 13 from vector only expressing canine keratinocytes; Lanes 2, 8, and 14 from CPV2 E6 expressing canine keratinocytes; Lanes 3, 9, and 15 from CPV2 E7 expressing canine keratinocytes; Lanes 4, 10, and 16 from CPV2 E6/E7 expressing canine keratinocytes; Lanes 5, 11, and 17 from RNA without reverse transcriptase (RT-); and Lanes 6, 12, and 18 are no template controls.
