# Supplementary material for: Abrogation of Constitutive and Induced Type I and Type III Interferons and Interferon-Stimulated Genes in Keratinocytes by Canine Papillomavirus 2 E6 and E7
Source: Viruses. 2020 Jun 23;12(6):677. doi: 10.3390/v12060677 (PMC7354437; doi:10.3390/v12060677)
Supplement: Supplementary file 1 [file viruses-12-00677-s001.zip › viruses-809279 final supplementary/Figure S2.pdf]

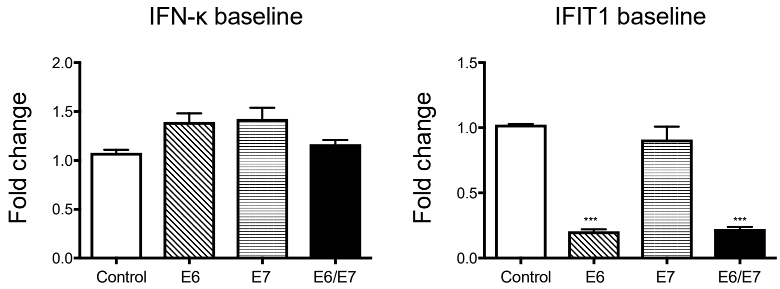

Supplemental Fig. 2. Constitutive mRNA expression of interferon (IFN)-κ and IFIT1 in canine papillomavirus (CPV)2 E6, E7, and E6/E7 expressing canine keratinocytes. Canine keratinocytes were generated that express vector only, CPV2 E6, CPV2 E7, or CPV2 E6/E7 using retrovirus stocks prepared by transfecting the retrovirus packaging cell line SD3443 cells with vector only, CPV2 E6, CPV2 E7, or CPV2 E6/E7 retrovirus constructs using Fugene as specified by the manufacturer and as previously described [24]. Primary canine keratinocytes expressing vector only, CPV2 E6, CPV2 E7, or CPV2 E6/E7 were cultured for 48 hours before mRNA expression of IFN-κ and IFIT1 were assessed by quantitative RT-PCR. Resulting Cq values were normalized to the Cq value of the reference gene and calibrated to mRNA expression in vector only control. mRNA expression of IFN-κ was not significantly altered in any of the cells. mRNA expression of IFIT1 was significantly diminished in canine keratinocyte expressing CPV2 E6 and CPV2 E6/E7. Experiments were performed in triplicate and repeated in two independent experiments. Results are expressed as mean +/- SEM. ***$p<0.001$. IFIT1, IFN-Induced Protein with Tetratricopeptide Repeats 1.
